# Comorbidities and clinical features related to severe outcomes among COVID-19 cases in Selangor, Malaysia

**DOI:** 10.5365/wpsar.2020.11.3.007

**Published:** 2021-02-16

**Authors:** Wan Shakira Rodzlan Hasani, Shubash Shander Ganapathy, Chong Zhuo Lin, Halizah Mat Rifin, Mohammad Nazarudin Bahari, Muhammad Haikal Ghazali, Noor Aliza Lodz, Muhd Hafizuddin Taufik Ramli, Nur Liana Ab Majid, Miaw Yn Jane Ling, Muhammad Fadhli Mohd Yusoff, Noor Ani Ahmad, Anita Suleiman, Ahmad Faudzi Yusoff, Venugopalan Balan, Sha’ari Ngadiman

**Affiliations:** a Institute for Public Health, National Institutes of Health, Ministry of Health Malaysia, Setia Alam, Selangor, Malaysia.; b Selangor State Health Department, Ministry of Health Malaysia, Shah Alam, Selangor, Malaysia.; c Disease Control Division, Ministry of Health Malaysia, WP Putrajaya, Malaysia.; d Institute for Medical Research, National Institutes of Health, Ministry of Health Malaysia, Setia Alam, Selangor, Malaysia.

## Abstract

**Background:**

Pre-existing comorbidities can predict severe disease requiring intensive care unit (ICU) admission among COVID-19 cases. We compared comorbidities, clinical features and other predictive factors between COVID-19 patients requiring ICU admission for intubation/mechanical ventilation and all other COVID-19 cases in Selangor, Malaysia.

**Method:**

Field data collected during the COVID-19 outbreak in Selangor, Malaysia, up to 13 April 2020 were used, comprising socio-demographic characteristics, comorbidities and presenting symptoms of COVID-19 cases. ICU admission was determined from medical records. Multiple logistic regression analysis was performed to identify factors associated with ICU admission requiring intubation/mechanical ventilation among COVID-19 cases.

**Results:**

A total of 1287 COVID-19-positive cases were included for analysis. The most common comorbidities were hypertension (15.5%) and diabetes (11.0%). More than one third of cases presented with fever (43.8%) or cough (37.1%). Of the 25 cases that required intubation/mechanical ventilation, 68.0% had hypertension, 88.0% had fever, 40.0% had dyspnoea and 44.0% were lethargic. Multivariate regression showed that cases that required intubation/mechanical ventilation had significantly higher odds of being older (aged ^3^60 years) [adjusted odds ratio (aOR) = 3.9] and having hypertension (aOR = 5.7), fever (aOR = 9.8), dyspnoea (aOR = 9.6) or lethargy (aOR = 7.9) than cases that did not require intubation/mechanical ventilation.

**Conclusion:**

The COVID-19 cases in Selangor, Malaysia requiring intubation/mechanical ventilation were significantly older, with a higher proportion of hypertension and symptoms of fever, dyspnoea and lethargy. These risk factors have been reported previously for severe COVID-19 cases, and highlight the role that ageing and underlying comorbidities play in severe outcomes to respiratory disease.

Coronavirus disease 2019 (COVID-19) is an infectious disease caused by severe acute respiratory syndrome coronavirus 2 (SARS-CoV-2). The infection was first detected in Wuhan, China, and has since spread across mainland China (*1*) and all over the world. COVID-19 is the third coronavirus infection that has spread widely, after SARS and Middle East respiratory syndrome (MERS). (*2*) On 11 March 2020, WHO declared COVID-19 a pandemic. (*3*) As of 14 April 2020, COVID-19 had led to 1 848 439 diagnosed cases and 117 217 deaths worldwide. (*4*) Up to 14 April 2020, Malaysia has had a total of 4987 infected people and 82 deaths. (*5*)

With the increasing numbers of confirmed cases and fatalities due to COVID-19, underlying comorbidities such as cardiovascular diseases and immune deficiency, especially among elderly patients, have been shown to be predictors of severe disease outcomes and poor prognoses in COVID-19 patients. (*6*-*8*) Severe cases of COVID-19 often require admission to an intensive care unit (ICU). In China, about 15% of patients developed severe pneumonia, and about 6% required non-invasive or invasive ventilatory support. (*9*)

Early identification of the risk factors of severe COVID-19 disease requiring intensive care in hospital would be helpful for managing hospital admissions. Studies in China and Italy suggest that the risk factors for severe COVID-19 include underlying comorbidities. (*10*, *11*) Therefore, we compared the comorbidities, clinical features and other predictive factors of COVID-19 patients requiring admission to ICU for intubation/mechanical ventilation with all other COVID-19 cases in Selangor, Malaysia.

## Methods

In this retrospective study, data were collected during a COVID-19 outbreak in Selangor, Malaysia, a state on the west coast of peninsular Malaysia. At the time of the study, Selangor had recorded the highest number of COVID-19 cases in the country, with the first case reported on 4 February 2020. The analysis included all laboratory-confirmed cases in the state up to 13 April 2020. Cases were confirmed by reverse transcriptase polymerase chain reaction (RT–PCR) testing. (*12*)

Descriptive methods were used to analyse socio-demographic characteristics, comorbidities, clinical presentation and the proportion of ICU admissions requiring intubation/mechanical ventilation. Clinical and socio-demographic characteristics were derived from case investigation reports obtained from district health offices in charge of each patient. The symptoms were self-reported during telephone interviews of cases by district health officers upon notification of a positive COVID-19 case. Admission to ICU for intubation/mechanical ventilation was verified from hospital records.

Multiple logistic regression analysis was performed to identify factors associated with intubation/mechanical ventilation among COVID-19 cases. The outcome variable was ICU admission requiring intubation/mechanical ventilation. The predictor variables included a history of hypertension, diabetes, heart disease, chronic respiratory disease (including asthma, chronic obstructive pulmonary disease and emphysema), cancer and kidney disease and the main symptoms of COVID-19, such as fever, cough, dyspnoea, lethargy, arthralgia, myalgia, headache and diarrhoea. Variables with *P*-values < 0.25, based on Wald χ2 statistics in univariate analysis, (*13*) as well as those variables considered to be biologically plausible, were selected for the multivariate analysis.

The final model included sex, age, hypertension, diabetes, heart disease, fever, cough, dyspnoea and lethargy. The univariate test *P*-value cut-off was set at 0.25 because the usual level of 0.05 may fail to identify variables known to be important. (*14*) A backward selection method was used to select variables. Starting with all candidate variables, the least significant effect for the model was removed, and the process was repeated until no further variables could be deleted without a statistically significant loss of fit. After this process, only age, hypertension, fever, dyspnoea and lethargy were significant in the multivariate model. To avoid over-fitting the model, only these five variables were included. Possible multicollinearity and all possible two-way interaction terms were checked one by one with main effect. Goodness-of-fit statistics were used to assess the fit of the regression model against actual outcomes. Two-sided *P*-values < 0.05 were considered statistically significant. Statistical Package Social Sciences (SPSS) statistical software version 24 was used for the analysis.

The study was conducted in accordance with the Declaration of Helsinki and ethical approval was obtained from the National Medical Research Registry, Ministry of Health Malaysia (registration number NMRR-20–720–54598). The requirement for written informed consent was waived given the context of an emerging infectious disease.

## Results

In total, 1287 laboratory-confirmed cases of COVID-19 in Selangor were included in the analysis. Of these, 750 patients (58.3%) were male and most (74.0%) were of Malay ethnicity. The median age was 36 years, and the highest percentage of cases were in people aged 18–29 years. The most commonly reported comorbidities were hypertension (15.5%) and diabetes (11.0%). More than one third of cases presented with fever (43.8%) or cough (37.1%); only 5.5% experienced dyspnoea, and 6.1% were lethargic (**Table 1**).

**Table 1:** Socio-demographic characteristics, comorbidity and clinical presentation of COVID-19- positive cases in Selangor (*n* = 1287)

Characteristic		COVID-19-positive cases
**Sex, *n*(%)**		**-**
-	Male	750 (58.3)
-	**Female**	**537 (41.7)**
**Age (years)**		**-**
**-**	**Median (IQR)**	**36.0 (30.0)**
**-**	**Mean (SD)**	**38.8 (18.2)**
Age groups, ***n*(%)**		**-**
**-**	** < 18**	**116 (9.0)**
**-**	**18–29**	**366 (28.4)**
**-**	**30–39**	**239 (18.6)**
**-**	**40–49**	**151 (11.7)**
**-**	**50–59**	**214 (16.6)**
**-**	**^3^60**	**201 (15.6)**
Ethnicity, ***n*(%)**		**-**
**-**	**Malay**	**952 (74.0)**
**-**	**Chinese**	**118 (9.2)**
**-**	**Indian**	**43 (3.3)**
**-**	**Other**	**174 (13.5)**
Nationality, ***n*(%)**		**-**
**-**	**Malaysian**	**1122 (87.2)**
**-**	**Non-Malaysian**	**165 (12.8)**
Comorbid conditions, ***n*(%)**		**-**
**-**	**Hypertension**	**200 (15.5)**
**-**	**Diabetes**	**141 (11.0)**
**-**	**Heart disease or other problem**	**50 (3.9)**
**-**	**Chronic respiratory disease**	**40 (3.1)**
-	**Chronic kidney disease**	**18 (1.4)**
**-**	**Cancer**	**7 (0.5)**
**-**	**Current smoker**	**57 (4.4)**
Symptoms, ***n*(%)**		**-**
**-**	**Fever**	**564 (43.8)**
**-**	**Cough**	**477 (37.1)**
**-**	**Lethargy**	**78 (6.1)**
**-**	**Dyspnoea**	**71 (5.5)**
**-**	**Headache**	**71 (5.5)**
**-**	**Myalgia**	**53 (4.1)**
-	**Diarrhoea**	**41 (3.3)**
**-**	**Arthralgia**	**31 (2.4)**
Hospital admission (*n* = 1 156), ***n*(%)**		**-**
**-**	**Intubated (invasive ventilator support)**	**25 (2.2)**
**-**	**Not intubated**	**1131 (97.9)**

Of the 1156 patients who were hospitalized, 25 (2.2%) were admitted to the ICU and required intubation/mechanical ventilation. Of these 25 cases, 14 were aged (*3*)60 years, 17 had hypertension, 10 had diabetes, 22 presented with fever, 14 with cough, 10 with dyspnoea and 11 with lethargy (**Table 2**).

**Table 2:** Numbers of intubated cases of COVID-19 by socio-demographic, NCD comorbidity and clinical presentation

Variables		Intubated (invasive mechanical ventilation) (*n* = 25)	Not intubated (*n* = 1 262)
**Sex, *n*(%)**		**-**	
-	Male	18 (72.9)	732 (58.0)
** -**	**Female**	**7 (28.0)**	**530 (42.0)**
**Age group, *n*(%)**		**-**	
** -**	** < 60**	**11 (44.0)**	**1075 (85.2)**
** -**	**^3^60**	**14 (56.0)**	**187 (14.8)**
**Comorbid conditions, *n*(%)**		**-**	
** -**	**Hypertension**	**17 (68.0)**	**183 (14.5)**
** -**	**Diabetes**	**10 (40.0)**	**131 (10.4)**
**-**	**Heart disease**	**4 (16.0)**	**46 (3.6)**
** -**	**Chronic respiratory disease**	**0 (0.0)**	**40 (3.2)**
** -**	**Chronic kidney disease**	**3 (12.0)**	**15 (1.2)**
** -**	**Cancer**	**0 (0.0)**	**7 (0.6)**
** -**	**Current smoker**	**1 (4.0)**	**56 (4.4)**
**Symptoms, *n*(%)**		**-**	
** -**	**Fever**	**22 (88.0)**	**542 (42.9)**
** -**	**Cough**	**14 (56.0)**	**463 (36.7)**
** -**	**Lethargy**	**11 (44.0)**	**67 (5.3)**
** -**	**Dyspnoea**	**10 (40.0)**	**61 (4.8)**
** -**	**Diarrhoea**	**3 (12.0)**	**38 (3.0)**
** -**	**Arthralgia**	**1 (4.0)**	**30 (2.4)**
** -**	**Myalgia**	**1 (4.0)**	**52 (4.1)**
** -**	**Headache**	**0 (0.0)**	**71 (5.6)**

The final multivariate model demonstrated that the odds of COVID-19 cases that required intubation/mechanical ventilation being older ((*3*)60 years) were 4.2 times (aOR: 4.24, 95% CI: 1.59–11.34) higher than the odds of all other cases being older, after controlling for sex, comorbidities and presenting symptoms. COVID-19 cases that required intubation/mechanical ventilation also had 6.0 times higher odds of having underlying hypertension (aOR: 5.97, 95% CI: 2.27–15.72) and presenting with the symptoms of fever (aOR: 7.91, 95% CI: 2.18–28.73), dyspnoea (aOR: 8.47, 95% CI: 3.08–23.29) or lethargy (aOR: 7.57, 95% CI: 2.89–19.86), compared with the odds for these risk factors in all other cases (**Table 3**). When age was used as a continuous variable in the same regression model, every 1-year increase in age increased the odds of requiring intubation/mechanical ventilation by 8% (aOR: 1.08, 95% CI: 1.03–1.12).

**Table 3:** Factors associated with intubation among positive COVID-19 cases in a binary logistic regression model (*n* = 1287)

Risk factor		Simple logistic regression			Multiple logistic regression		
		*b*	Crude OR (95% CI)	*P*	*b*	Adjusted OR* (95% CI)	*P*
**Sex**							
-	Male	-	1	-	-	-	-
**-**	**Female**	–**0.62**	**0.54 (0.22–1.30)**	**0.166**	**-**	**-**	**-**
**Age group (years)**							
**-**	** < 60**	**-**	**1**	**-**	**-**	**1**	**-**
**-**	**^3^ 60**	**1.99**	**7.32 (3.27–16.36)**	** < 0.001**	**1.45**	**4.24 (1.59–11.34)**	**0.004**
**Hypertension**							
**-**	**No**	**-**	**1**	**-**	**-**	**1**	**-**
**-**	**Yes**	**2.53**	**12.53 (5.33–29.46)**	** < 0.001**	**1.79**	**5.97 (2.27–15.72)**	** < 0.001**
**Diabetes**							
**-**	**No**	**-**	**1**	**-**	**-**	**1**	**-**
**-**	**Yes**	**1.75**	**5.76 (2.53–13.07)**	** < 0.001**	**-**	**-**	**-**
**Heart disease**							
**-**	**No**	**-**	**1**	**-**	**-**	**1**	**-**
**-**	**Yes**	**1.62**	**5.04 (1.66–15.26)**	**0.004**	**-**	**-**	**-**
**Fever at presentation**							
**-**	**No**	**-**	**1**	**-**	**-**	**1**	**-**
**-**	**Yes**	**2.28**	**9.74 (2.90–32.72)**	** < 0.001**	**2.07**	**7.91 (2.18–28.73)**	**0.002**
**Cough at presentation**							
**-**	**No**	**-**	**1**	**-**	**-**	**1**	**-**
**-**	**Yes**	**0.787**	**2.20 (0.99–4.88)**	**0.053**	**-**	**-**	**-**
**Dyspnoea at presentation**							
**-**	**No**	**-**	**1**	**-**	**-**	**1**	**-**
**-**	**Yes**	**2.58**	**13.13 (5.66–30.42)**	** < 0.001**	**2.14**	**8.47 (3.08–23.29)**	** < 0.001**
**Lethargy at presentation**							
**-**	**No**	**-**	**1**	**-**	**-**	**1**	**-**
**-**	**Yes**	**2.64**	**14.01 (6.13–32.05)**	** < 0.001**	**2.03**	**7.57 (2.89–19.86)**	** < 0.001**

* Backward multiple logistic regression was applied. Multicollinearity and interactions were checked and not found. Hosmer Lameshow test *P*  **= 0.808, classification table (overall correctly classified percentage = 98.0%) and area under ROC curve = 94.3% were used to check model fitness.**

Univariable analyses were also conducted for cancer, chronic kidney disease, current smoker, chronic respiratory disease and symptoms at presentation such as diarrhoea, arthralgia, myalgia and headache. The results are not presented in the table because small cell sizes did not give meaningful ORs and CIs.

Univariable analyses were also conducted for cancer, chronic kidney disease, current smoker, chronic respiratory disease and symptoms at presentation such as diarrhoea, arthralgia, myalgia and headache. The results are not presented in the table because the small sample sizes did not give meaningful ORs and CIs.

## Discussion

In this study, the proportion of COVID-19 cases requiring intubation/mechanical ventilation in Selangor, Malaysia (2.2%) was similar to studies in China (2.3–3.0%) (*10*, *15*) but lower than that in the United States of America (20.2–22.3%). (*16*, *17*) These differences may be due to differences in guidelines for intubation and mechanical ventilation as well as ICU bed capacity. We also found that underlying hypertension and diabetes were the most common comorbidities in all COVID-19 cases, consistent with the findings in Wuhan (*18*) and in a meta-analysis of the prevalence of comorbidities in COVID-19 patients. (*19*) Bornstein et al. (*20*) reported that hypertension and type-II diabetes were the most common comorbidities in infected COVID-19 patients, due to metabolic inflammation caused by the infection, which compromises the immune system. Diabetes and hypertension were also reported as the most common comorbidities with other coronaviruses, such as SARS-CoV and MERS-CoV. (*21*)

Older age and underlying comorbidities are predictors of severe outcomes in viral infections generally, (*22*, *23*) and we found that the proportion of COVID-19 patients who required intubation/mechanical ventilation increased with age. The regression model showed that the odds of requiring intubation/mechanical ventilation was 4.2 times higher for adults aged (*3*)60 years after controlling for comorbidities and presenting symptoms. As in other studies, the risk for a severe outcome is higher for older people. Data from China indicate that older adults with severe underlying health conditions are at higher risk for severe COVID-19-associated illness and death. (*24*) Reports from Italy also suggested that the risk factors for severe disease include older age and the presence of at least one underlying health condition among COVID-19 cases. (*11*)

Preliminary findings from the United States of America suggested that people with underlying health conditions are at higher risk for severe disease from COVID-19. (*25*) A study in China showed that almost 70% of COVID-19 patients who were admitted to an ICU had comorbidities. (*26*) Our study shows that COVID-19 patients with underlying hypertension contributed to a high percentage of ICU admissions requiring intubation/ mechanical ventilation. Cases that required intubation/mechanical ventilation also had six times the odds of having underlying hypertension after adjustment for age, other comorbidities and clinical presentation. Hypertension was the most common comorbidity that predicted a poor prognosis in patients with COVID-19. In a systematic review and meta-analysis by Yang et al., (*26*) the pooled odds of hypertension in patients with severe, as compared with non-severe disease, was 2.36 (95% CI: 1.46–3.83).

Other than age and underlying hypertension, the presenting symptoms of COVID-19 infection also predict a severe outcome. As in other studies, the most common presenting symptoms in this study were fever, cough, dyspnoea and lethargy. (*10*, *15*, *18*, *27*, *28*) Our findings indicate that symptomatic COVID-19 patients with fever, dyspnoea and lethargy have a strong, significant risk for intubation/mechanical ventilation. Li et al. (*29*) demonstrated significant differences in clinical symptoms and computed tomography scan manifestation between patients with or without severe or critical COVID-19 after control for age and comorbidities. This finding is important for clinicians in stratifying risk for their patients according to presenting symptoms. Although dyspnoea is a known risk factor for intubation/mechanical ventilation, patients presenting with fever or lethargy should also be closely monitored.

Our study has some notable limitations. First, the number of cases requiring intubation/mechanical ventilation was small at 25 cases (2.2%). Furthermore, the data were derived from a report from a single state in Malaysia and may not represent the national population. Data on ICU admissions, comorbidities and outcomes were missing for < 20% of patients. Despite these limitations, our results are consistent with previous studies of COVID-19 cases.

## Conclusion

COVID-19 cases that were intubated and ventilated had higher odds of being aged (*3*)60 years, having hypertension and presenting with fever, dyspnoea or lethargy compared with all other COVID-19 cases. Older people and those with comorbidities such as hypertension should be prioritized for hospital care as they are more vulnerable to severe disease and progression when infected with SARS-CoV-2.
